# Biology and Biomechanics of the Heart Valve Extracellular Matrix

**DOI:** 10.3390/jcdd7040057

**Published:** 2020-12-16

**Authors:** Karthik M. Kodigepalli, Kaitlyn Thatcher, Toni West, Daniel P. Howsmon, Frederick J. Schoen, Michael S. Sacks, Christopher K. Breuer, Joy Lincoln

**Affiliations:** 1 Department of Pediatrics, Medical College of Wisconsin, Milwaukee, WI 53226, USA; mkodigepalli@mcw.edu (K.M.K.); kthatcher@mcw.edu (K.T.); 2 Center for Cardiovascular Simulation, Institute for Computational Engineering and Sciences and Department of Biomedical Engineering, The University of Texas at Austin, Austin, TX 78712, USA; toni.west@austin.utexas.edu (T.W.); daniel.howsmon@utexas.edu (D.P.H.); msacks@oden.utexas.edu (M.S.S.); 3 Department of Pathology, Brigham and Women’s Hospital and Harvard Medical School, Boston, MA 02115, USA; fschoen@bwh.harvard.edu; 4 Tissue Engineering and Surgical Research, The Research Institute at Nationwide Children’s Hospital, Columbus, OH 43205, USA; christopher.breuer@nationwidechildrens.org; 5 Department of Pediatric Surgery, Nationwide Children’s Hospital, Columbus, OH 43205, USA; 6 The Herma Heart Institute, Section of Pediatric Cardiology, Children’s Wisconsin, Milwaukee, WI 53226, USA

**Keywords:** heart valve, extracellular matrix, collagen, proteoglycan, elastin, connective tissue disorders

## Abstract

Heart valves are dynamic structures that, in the average human, open and close over 100,000 times per day, and 3 × 10^9^ times per lifetime to maintain unidirectional blood flow. Efficient, coordinated movement of the valve structures during the cardiac cycle is mediated by the intricate and sophisticated network of extracellular matrix (ECM) components that provide the necessary biomechanical properties to meet these mechanical demands. Organized in layers that accommodate passive functional movements of the valve leaflets, heart valve ECM is synthesized during embryonic development, and remodeled and maintained by resident cells throughout life. The failure of ECM organization compromises biomechanical function, and may lead to obstruction or leaking, which if left untreated can lead to heart failure. At present, effective treatment for heart valve dysfunction is limited and frequently ends with surgical repair or replacement, which comes with insuperable complications for many high-risk patients including aged and pediatric populations. Therefore, there is a critical need to fully appreciate the pathobiology of biomechanical valve failure in order to develop better, alternative therapies. To date, the majority of studies have focused on delineating valve disease mechanisms at the cellular level, namely the interstitial and endothelial lineages. However, less focus has been on the ECM, shown previously in other systems, to be a promising mechanism-inspired therapeutic target. Here, we highlight and review the biology and biomechanical contributions of key components of the heart valve ECM. Furthermore, we discuss how human diseases, including connective tissue disorders lead to aberrations in the abundance, organization and quality of these matrix proteins, resulting in instability of the valve infrastructure and gross functional impairment.

## 1. Introduction

There are two sets of cardiac valves: the mitral and tricuspid atrioventricular valves that separate the atria from the ventricles; and the aortic and pulmonic semilunar valves that separate the ventricles from the great arteries. Although the functional demand for each valve set is similar, their anatomies are different. The atrioventricular valves consist of two (mitral) or three (tricuspid) leaflets, with external supporting chordae tendineae that attach the leaflet to papillary muscles within the ventricles (reviewed [1]). In contrast, the semilunar valves, located at the base of the aorta and pulmonary trunk, are comprised of three leaflets termed cusps, and lack an external chordae and papillary muscles, although a unique internal support structure has been described [2]. The heart valves open and close over 100,000 times per day, 40 million times per year, and over 3 billion times in a 75 year lifetime to maintain unidirectional blood flow during the cardiac cycle. In diastole, the papillary muscles are relaxed and high pressure in the atrium causes opening of the mitral (left) and tricuspid (right) valve leaflets to promote blood flow into the respective ventricle. Once ventricular pressure increases during diastole, the chordae ‘pull’ the atrioventricular valve leaflets closed and maintain coaptation to prevent eversion of the valve leaflets into the atria. As the ventricle contracts, blood exits through the now open semilunar valves and the ventricle relaxes to begin the cycle again. Therefore, throughout the cardiac cycle heart valve structures are exposed to constant changes in hemodynamic force as a result of pressure differences between systole to diastole. The valves experience mechanical stresses which include: (i) tension, when the valve is in the closed position; (ii) flexure, which occurs during closing and opening motions; and (iii) shear stress, experienced by contact with blood flow in the open position [3]. Healthy heart valves withstand these stresses by continuously maintaining, and adapting an intricate yet highly ordered connective tissue system within the valvular structures [1].

**Figure 1:**
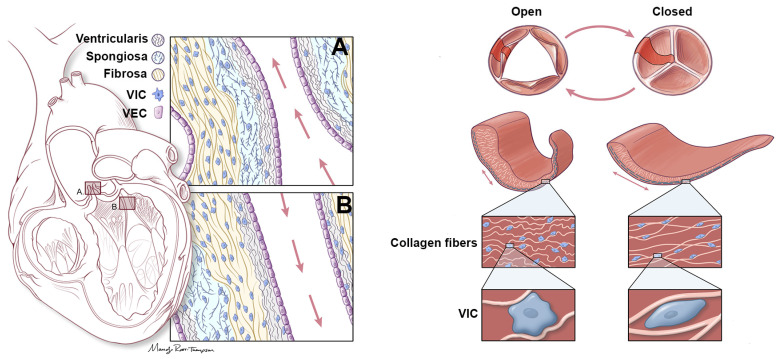
Representation of aortic and mitral valve structure. (Left) Aortic (**A**) and Mitral (**B**) valve structures to show organization of three ECM layers, including the ventricularis (elastin), spongiosa (PGs-GAGs) and fibrosa (collagens). Each layer is arranged according to blood flow as indicated by red arrows (ventricularis/atrialis closest to blood flow). Overlying the valve leaflets (mitral) or cusps (aortic) is a single layer of valve endothelial cells (VECs, purple), while a population of valve interstitial cells (VICs, blue) are embedded within the core. (Right) Representation of the aortic valve indicating coordinated rearrangement of the ECM fibers, and elongation of the VICs during systole (open) and diastole (closed).

The predominant biomechanical function of the valve is attributed to a highly organized network of extracellular matrix (ECM) components [4]. In addition to supporting structure-function relationships of the valve, the ECM also serves as a pool for signaling molecules to facilitate many biological processes [5]. In healthy valves, the ECM is organized into three highly organized layers (potentially four in humans) that are arranged according to blood flow (Figure 1). The fibrosa layer is located close to the outflow surface and is composed of densely aligned collagen fibers which provide the primary strength to the valves. The ventricularis layer is located on the opposite inflow surface and its high content of elastin facilitates stretch and retraction during the cardiac cycle. Sandwiched in between is the spongiosa layer, described as a central core of loose connective tissue high in proteoglycans (PGs)-glycosaminoglycans (GAGs)*,* which accommodates the relative motions of the neighboring layers. The ECM components of the valve leaflet are populated by valvular interstitial cells (VICs) and encapsulated by an overlying single layer of valve endothelial cells (VECs) (Figure 1). Based on the necessity of the ECM for cycle to cycle biomechanical function, it follows that the ongoing quantity, quality and architecture of the valvular ECM, particularly collagen, elastin, and PGs-GAGs, also determines the adaptability, and long-term (lifetime) durability of the valve.

Recent evidence indicates that the ECM layers are tightly bound and do not slide with respect to each other [6]. Therefore, while each layer of the heart valve is histologically distinct, each acts independently as a functionally graded material with unique properties that vary continuously over the cross-section of the leaflet [6,7]. Moreover, the layered structure spatially varies considerably between leaflets, and within the same leaflet. Histological studies have shown that the overall valve structure, composition and organization of the valve ECM is conserved across many species with more apparent order being observed in larger animals [2]. Clearly, the ECM is critical for valve structure-function relationships, and any imbalance to this is detrimental. Similar to the game “Jenga”, removal or disturbances of a wooden block can result in complete disassembly of the overall structure, and this is often experienced and exaggerated in valve disease states.

Homeostasis of the valve ECM is regulated by a heterogeneous population of VICs that, in healthy adults, are phenotypically similar to fibroblasts and mediate physiological ECM remodeling within the leaflet/cusp in response to the normal ‘wear and tear’ with aging [8]. This is achieved through a balanced secretion of matrix degradation enzymes including matrix metalloproteinases (MMPs) and their inhibitors (TIMPs), and deposition of structural ECM components within the layers [9,10]. Therefore, the VIC population plays a critical role in preserving the architecture of the valve for functional biomechanics. In addition to the VICs, the valve leaflet or cusp is encapsulated by a single cell layer of VECs that primarily form a tight and functional barrier between the blood and the inner valve tissue, thereby, protecting it against the physical and mechanical stress of the hemodynamic environment, and preventing excess infiltration of circulating risk factors and inflammatory cells [11,12]. In addition, VECs have been shown to molecularly communicate with underlying VICs to regulate their phenotype [13,14]. Therefore, in addition to VICs, integrity and function of the valve endothelium is important for supporting structure-function relationships throughout life.

Therefore, the valve leaflets are highly complex and dynamic structures characterized by sophisticated architectural networks formed by the diverse ECM components. Over the years, there has been a focus on understanding the cell populations of the valve, and while the field has advanced in this area, the ECM has been less frequently highlighted. Therefore, this review aims to highlight the critical role of the ECM in establishing and maintaining the “lub-dub” during the cardiac cycle by highlighting the predominant compositions, their organization, and function in health and valve pathology.

## 2. Extracellular Matrix Components

### 2.1. Collagens in Heart Valves

The collagen superfamily comprises 28 members (Collagen I through XXVIII) and these can be divided into several subfamilies, based on their supramolecular assemblies, such as fibrillar collagens, fibril-associated collagens with interrupted triple helices (FACIT), anchoring collagen fibrils, and collagen networks [15]. In heart valves, collagen is the major stress-bearing component and makes up almost 90% of the valve ECM. They are predominantly localized to the fibrosa layer where they provide stiffness and strength, and serve to maintain apposition of the cusps without prolapse into the left ventricle [5]. Several expression studies in mice have identified various collagen types within the heart valve leaflets [16] which include fibril-forming collagens of type I, II [17], III [18], V [19] and XI [20], and network/beaded-filament forming collagens of type IV [20] and VI [20,21]. In addition, solubilization studies of healthy human mitral valves reported the relative amounts of several collagen types and estimated their abundances to be approximately 74% for type I, 24% for type III, and 2% for type V [22,23,24].

Collagen fiber arrangement within the three layers of the leaflets, but predominantly in the fibrosa, facilitates the continued cyclic deformation. In healthy valves, collagen fibers are preferentially aligned along the circumferential axis. Free collagen content within the valves is low, signifying that collagen secreted by VICs is quickly integrated into fibrils [25,26]. MMPs enzymatically remove the N- and C- terminal ends of the free collagen so that collagen polymerizes end-to-end to form long fibrils. This degrative activity of MMPs is held in check by their inhibitors (TIMPs) in healthy valves, minimizing further ECM degradation [27]. Although the free collagen polymerizes end-to-end, the angle between two pieces is kinked, resulting in a repeating dark-light banding pattern of collagen fibrils which is measured as the D-period. Physiological biaxial tension does not increase the D-period, denoting that these tiny kinks are not pulled straight in the valves of healthy hearts. At higher than physiological biaxial tension, the D-period increases linearly with force applied. This denotes that an individual collagen fibril structure does not have an intrinsic viscoelastic behavior under physiologic conditions [28,29].

Collagen fiber-level characteristics allow the valve to be stretched repeatedly. In healthy valves, approximately 605 parallel collagen fibrils crosslink into a single collagen fiber. Individual fibrils can be resolved under unloaded conditions, but upon physiological levels of biaxial stretch, they are packed together tightly and cannot be resolved even with electron microscopy [30]. Collagen fibers do not stay in a rigid straight line when not being stretched; instead they relax into slightly different angles from the primary circumferential axis and form wavy patter called collagen crimp. This wave-like pattern that is produced in parallel collagen fibers acts as an initial shock absorber during biaxial stretch. When the valve is initially stretched, the crimped collagen straightens out and rotates, allowing the leaflet to coapt and form a seal. When the valve is subsequently unloaded, this process is reversed. This property imparted by collagen onto the valve leaflet tissue is a major way the valve functions [25]. It should be noted that this process occurs with minimal fiber-fiber or fiber-matrix interactions [29], and demonstrates how nature has evolved a highly mechanically-compliant structure primarily containing very stiff collagen fibers [31,32,33]. Collagen fibers in heart valves, like other planar collagenous tissues, follow the principles of the affine kinematic model, where a collagen fiber when under biaxial stretch, deforms as a single, homogenous fiber-level unit that is approximately 1mm in size. In general, collagen fibers align well with the circumferential axis [29]. However, there is more variation in the half of the leaflet that interacts with the shear flow of blood (21° splay in the ventricularis of the aortic valve) than in the back half of the leaflet (14° splay in the fibrosa of the aortic valve), with a gradual transition of splay in the spongiosa region [7,34]. This suggests that the ventricularis collagen can experience more stretch than that of the fibrosa, while recruitment strains of the different layers are invariant [29]. Indeed, modeling of the experimental data identifies that circumferential stress is much higher in the fibrosa than in the ventricularis of aortic valve leaflets [7].

### 2.2. Proteoglycans in Heart Valves

PGs consist of a core protein, and one or more covalently attached GAGs. They can be classified into several groups based on their distribution and function. The major types are: (i) interstitial PGs that help in stabilizing and organizing the collagen fibers; (ii) the aggrecan family of PGs that includes aggrecan, versican, brevican and neurocan; (iii) secretory granule PGs expressed in cytoplasmic secretory granules; (iv) basement membrane PGs distributed in the basement membrane; and (v) small leucine-rich PGs (SLRPs) that include decorin, biglycan, fibromodulin and lumican [35]. As for GAGs, the major types that exist are hyaluronan, chondroitin sulfate (CS), dermatan sulphate (DS), keratan sulfates (KS), and heparan sulfate/heparin (HS). PGs are polar molecules that absorb water efficiently and are commonly interspersed within the collagen fibrils in ECMs and interact with collagens and other ECM components to form hydrated and stable matrices that are capable of enduring high compressive loads. Overall, the PGs provide space-filling and lubrication to the tissues to aide in withstanding compressive stress [5].

Hyaluronan is the main GAG in healthy adult human heart valves, although DS, CS and HS are also expressed at relatively lower levels (3–7%) [36,37]. At the level of PGs, versican is the most highly expressed, along with decorin and biglycan [37,38]. In addition to being structurally critical for cardiac development, versican provides essential lubrication to the valve tissues [39] and forms aggregates with hyaluronan to aide in loosening the matrix to facilitate cell proliferation and migration during valve development/remodeling [37,39]. Versican and hyaluronan are abundantly expressed in the spongiosa layer where they form a foam-like structure and bind water [5,40]. Versican has also been shown to potentially aide in tissue elasticity [39,41]. On the other hand, decorin and biglycan are found to be expressed predominantly in the flow-exposed ventricularis and atrialis layers of the healthy adult aortic, and mitral valve leaflets, respectively [38,42,43,44]. Decorin is also expressed in the fibrosa layer of the ECM where it plays a fundamental role in regulation of collagen fibril formation [43,45]. The free edges of the leaflet are also enriched with hyaluronan, 6-sulfated GAGs, and versican, while in the mitral position, the chordae tendineae contain an abundance of decorin, biglycan, and 4-sulfate GAGs [46].

Multiple studies have demonstrated that dysregulation of PGs and GAGs are commonly associated with valve abnormality and dysfunction (see Section 5) [38,43]. In mice, the incomplete cleavage of versican by ADAMTS5 leads to increased penetrance of bicuspid aortic and pulmonary valve disease [47]. In human calcific aortic valve tissue, versican, decorin, biglycan and hyaluronan are abundantly localized to the regions around the calcified nodules [44]. These proteins are also significantly overexpressed in human and canine myxomatous mitral valves, compared to control tissues [38,48]. In aging, the GAG-PG composition is markedly altered [43,49] with hyaluronan, decorin and biglycan being elevated in the fibrosa layer of mitral valve leaflets [43], and abnormal GAG sulfation patterns observed in a valve-region-dependent way [43,49].

### 2.3. Elastin in Heart Valves

Elastic fibers are a complex molecular assembly predominantly made up of an insoluble elastin protein core surrounded by a microfibrillar sheath [5,50,51]. Crosslinked elastin fibers have a remarkable capability to endure significant amounts of deformation under small loads and still recoil back to the original shape with minimal energy loss. Therefore, in the heart valves, elastic fibers largely within the ventricularis/atrialis layer are compactly organized in the form of continuous sheets and arranged along both radial and circumferential axes. This arrangement provides flexibility and stretch in response to the hemodynamic environment [5,42]. Although they do not contribute to the stiffness and strength of the valves, elastic fibers due to their manifold flexibility, significantly contribute to the valve flexion during cardiac cycle. During the valve closure phase, elastic fibers in the ventricularis endure significant and continual stretch. This extension also facilitates the unfolding of collagen fibers within the fibrosa layer during which they bear the load of entire leaflet. As the pressure load reduces, the elastic fibers recoil, which in turn retracts the valve leaflet back towards the annulus, and also facilitates the collagen fibers to re-fold and re-orient quickly for the next cycle [5,52].

### 2.4. Minor ECM Components of the Heart Valve

In addition to the major ECM components reviewed above, there are several minor components of ECM that play diverse functional roles within heart valves [5]. These include fibronectin, periostin, osteopontin, and laminins, among others. Other key components of the heart valve ECM family are the MMPs and TIMPs [5,53]. MMPs are the major proteinase enzymes that aide in the timely degradation of ECM, needed for tissue development, repair and remodeling. The expression and functions of the MMP family, consisting of 24 genes (*MMP1* to *24*) in various tissue, have been reviewed extensively elsewhere [54]. MMP activity is largely regulated via inhibition by endogenous inhibitors, TIMPs, which are critical for ECM remodeling and homeostasis. In addition to regulating the tissue structure via ECM degradation, MMPs can also chemically alter the ECM microenvironment, cell-matrix and cell-cell interactions [54]. ECM degradation results in release of growth factors bound to ECM molecules and make them available for the receptor mediated cell signaling. Additionally, MMPs have been implicated in several cellular processes including cell migration, inflammatory processes, apoptosis, and cell differentiation [54]. Correlative studies have suggested that MMPs play important roles in valve pathogenesis [55]. Stenotic and calcified aortic valve leaflets express elevated levels of MMP3, MMP9 and TIMP1 [55]. More specifically, MMP9 has been shown to localize at the calcific nodules, and to be associated with abnormal ECM remodeling, including disorganized collagen bundles, while MMP1 is thought to mediate inflammation-regulated ECM remodeling and calcific aortic valve stenosis [56,57]. Both MMP2 and MMP9 are mechanosensitive and their expression is increased following exposure to cyclic pressure and shear stress [58]. Therefore, their expression maybe exacerbated in stenotic disease associated with hemodynamic disturbances. In addition to calcification, The Schoen group were the first to report increased levels of MMP1, MMP3, MMP2 and MMP9 in excised myxomatous human valves at end stage disease [9], and similar findings were later reported in affected canines [59]. Additional studies have further shown a causative role for MMPs in mitral valve disease as determined following over expression of MMP2 in mice that exhibit phenotypes reminiscent of myxomatous degeneration [60]. These findings demonstrate the importance of a balanced MMP environment in regulating ECM remodeling in health and disease.

## 3. Signaling Pathways and ECM in Heart Valves

The cellular component of heart valves include VECs and VICs, and these cell populations play key roles in regulating the composition and organization of the ECM. Both cell types aide in valve development, remodeling and maintenance by providing critical autonomous and non-autonomous signaling mediators that arbitrate the balance of ECM turnover [5,61,62]. VICs, the most abundant of the two, and are primarily responsible for synthesizing and degrading the ECM [5,63]. VICs arise from multiple cell lineages during development, and remain a very heterogeneous population, which may reflect specific functions required for valvular homeostasis and function [10,64]. VIC phenotypes are still relatively underappreciated, but include: (i) quiescent (qVICs) that are at rest, with low expression of the myofibroblast marker smooth muscle α-actin (SMA), and maintain normal valve physiology; (ii) activated (aVICs) that more highly express SMA and regulate the valve pathobiological responses; in addition to (iii) progenitor VICs (pVICs) that play key roles in valve repair; and (iv) osteoblastic VICs (obVICs) that regulate osteogenesis and chondrogenesis [64]. Of these, myofibroblast-like aVICs are crucial for ECM metabolism and remodeling and are dysregulated in several valve diseases [9,10].

Physiological ECM remodeling and stratification are critical for establishing and maintaining adult valve structure and function [2] and the signaling pathways that regulate these processes have been identified and reviewed previously [5,63]. It is interesting to note that several of these ‘normal’ pathways are also implicated or dysregulated in valve diseases associated with disturbances in ECM homeostasis [5]. Transforming growth factor-β-1 (TGF-β1) signaling is critical for early stages of endothelial-to-mesenchymal-transformation during endocardial cushion development, but has also been implicated in valve disease [65]. TGF-β1 is well-known for inducing the activation of qVICs at least in human and large animal cultured cells, characterized by upregulation of SMA and subsequently promoting changes in ECM homeostasis [66,67]. In addition, previous studies have identified canonical TGF-β1 signaling in VICs as a regulatory pro-fibrotic pathway via SMAD regulation of collagen genes [66]. More recently, studies in cardiac fibroblasts and VICs have highlighted Scleraxis (Scx) as a key target of TGF-β1 [68,69]. In the valves, Scx is most highly expressed within the supporting structures, and associated with other tendinous matrix proteins, including Tenascin in regions of high mechanical demand [68]. While the function of Scx remains largely elucidated, published work in mouse cardiac fibroblasts has reported an important role in regulating the expression of many ECM components, including collagens, PGs, MMPs and TIMPs, and loss of function in mice severely attenuates CSPG and collagen expression profiles [63,70,71,72]. More importantly, *Scx* levels were found to be increased in VICs isolated from human myxomatous mitral valves suggesting a causative role in pathology associated with ECM disturbances [72]. Aside from the pro-fibrotic role of TGF-β1 signaling in heart valves, studies by the Lincoln Lab have identified endothelial-TGF-β1 paracrine signaling as anti-calcific through the positive regulation of Sox9 in VICs [14]. In addition to its anti-calcific role in VICs, Sox9 is required during early stages of valve development for proliferation of VIC precursor cells during endocardial cushion formation. However, at later stages it is necessary for the regulation of several ECM genes shared with chondrogenic development, including *ColII, Col1I, Aggrecan*, and *Cartilage-Link Protein* [63,70]. Together these studies suggest that TGF-β1-mediated signaling must be finely tuned, in order to maintain healthy homeostasis and prevent disturbances in VIC behavior and ECM organization.

## 4. Heart Valve ECM Function

### 4.1. Heart Valve Biomechanics

The intricate structure of the heart valve ECM facilitates its mechanically demanding physiological function. Valves constantly undergo deformation during each cardiac cycle [73], which is evident from echocardiography, as measured by angle of displacement during opening, changing length of the attached papillaries/chordae, the change in annulus/root shape [74,75], and quantification of forces imparted on the valve recently modeled from landmarking on 4-dimensional (4D) transesophageal echocardiograms [76]. The structure of the valve leaflets/cusps is uniquely designed to bend with their curvature while restricting bending against their curvature [77], allowing valves to maintain unidirectional blood flow while preventing regurgitation. Tissue-level deformation of the valve is also evident and this leads to deformation of resident VICs. Cytoplasmic and nuclear surface area-to-volume ratios of the VICs increase and cytoplasmic volume decreases when the leaflet goes from unloaded to loaded with physiological levels of biaxial stretch [30]. Understanding how heart valves are able to maintain and adapt their structure in the context of these mechanical deformations requires a multiscale approach.

### 4.2. VIC-Mediated Maintenance of ECM in Heart Valves

The exemplary durability of heart valves is largely made possible by the VICs by incorporating new collagen and other ECM molecules into the existing matrix, and remodeling them into the proper structural component. Compared to other dense connective tissues, heart valves are highly cellular, with ovine aortic valves having on average 2036 cells/mm^2^, and human aortic valves having on average 760 cells/mm^2^ [78]. Decellularization of ovine valves, but not human valves, increases biaxial areal strain and circumferential peak stretch [78], which suggests that VICs themselves and their interactions with ECM molecules directly affect response to physiological biaxial stretch in a threshold-dependent manner. In a healthy valve, VICs are generally ellipsoidal in shape [79], but they are highly dendritic and contain a large amount of cytoplasm [30]. This allows them to directly interact with 3-4 collagen fibers and the other associated surrounding ECM molecules [30]. During valvular disease states, VICs produce more ColI that is at more random angles [80]. Pregnancy-induced valve remodeling differs in modality from pathological remodeling [26] in that it is reversed after pregnancy [81], and thus, more closely resembles processes seen in development. During pregnancy, increases in forces due to non-pathological volume overload lead to a compensatory remodeling of the collagen matrix in the mitral valve, leading to valves that have a larger surface area and are thickened in processes mediated by VICs [82]. Initial increases in fibrosa thickness, total ColI, and the average angle of deflection of ColI fibrils indicate that new collagen is being laid down [25,26]. Then, the total and mature crosslinks of collagen are increased, while there is no change in immature crosslinks, as assessed by hydrothermal testing [81]. This indicates that the collagen network is formed into parallel fibers. The future implementation of genetic mouse models during pregnancy, will allow scientists to study the biomechanical properties that specific ECM molecule isoforms impart on the valves during developmental processes, since embryonic and neonatal mouse heart valves are difficult to study biomechanically because of their size.

**Figure 2:**
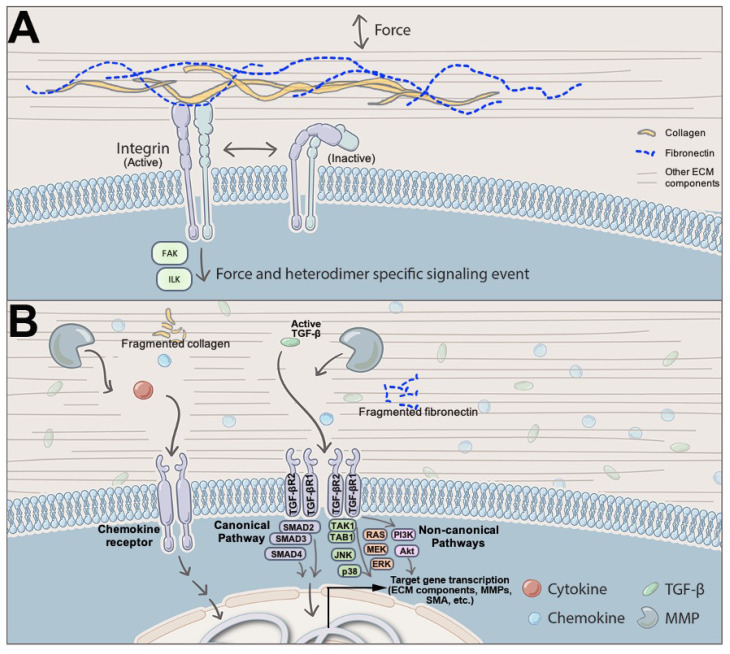
ECM-VIC Signaling Interactions. (**A**) Matricellular and mechanical signaling occurs in a force-, ligand-, and integrin heterodimer-specific manner. Once active, integrin heterodimer complex leads to activation of downstream pathways mediated by activation of signaling molecules, including focal adhesion kinase (FAK) and integrin-linked kinase (ILK). (**B**) During matricrine signaling in heart valves, metalloproteases (MMP) degrade structural components of the ECM, which allow signaling molecules such as cytokines, chemokines, and TGF-β to be released. Once activated by TGF-β, TGF-β receptors result in transcription of pro-fibrotic and pro-inflammatory genes via canonical or non-canonical TGF-β signaling cascade in VICs.

VIC contraction, stress fiber orientation, and connection to the ECM also play mechanically integral roles for the valve leaflet [83]. In a healthy valve, the VICs form focal adhesions with the surrounding ECM [84] and have a basal tonicity that contribute to the valve leaflet stiffness [85]. When the valve is under adverse stress, there is a mechanically-induced release of latent TGF-β from the ECM [86]. Subsequent TGF-β signaling activates the VICs, which is discussed (Figure 2). Upon activation, the cell remodels its interior cytoskeleton to contain larger quantities of SMA and stress fibers [3,87]. This increase in cytoskeletal components is correlated with cellular contraction of the VICs and stiffening of the ECM [85,88]. The cells contract, but maintain their focal adhesions, pulling and reorganizing the ECM [88]. It is important to note that stiff in vitro hydrogel systems that neither contain TGF-β nor other signaling molecules, can activate VICs, indicating that the increased forces between VICs and their surroundings alone can lead to VIC activation. Contraction, in turn, releases more latent TGF-β, which can form a positive feedback loop where VIC activation leads to further VIC activation, leading to disease. Recently 3D hydrogel cultures of VICs have been employed with some perplexing but reproducible results where stiffer gels lead to a decrease in VIC contractility [30,89,90]. This leads to speculate that the results in 3D culture are due to the lack of complexity of the hydrogel, which is present in native valves. For instance, implementing cyclical stretch [91], thereby increasing the number of binding peptides [89]; or adding electrospun microfibers [92], MMPs [88], ColI [93], methacrylated hyaluronic acid or methacrylated gelatin [93] to the 3D hydrogel produces a VIC phenotype more consistent to that seen in native valves. Although, tuning of the gels to different stiffnesses within most of these conditions has not been published.

In diseased valves, interior cytoskeleton remodeling continues. In cell culture, VICs can be differentiated into chondrocytes, osteoblasts, or osteoclasts, but it is more likely that osteoclasts differentiate from invading monocytes in a native valve [94]. Differentiation leads to a change in the types and ratio of both ECM and cytoskeletal molecules produced by the cells, changing the stiffness of the valve [95]. Some VICs apoptose and/or produce exosomes that can serve as seed for initial microcalcification, attaching to a wide variety of locations throughout the ECM, including collagen, charged areas of PGs exposed during ECM remodeling, integrins, and fibronectin. Osteoblasts then further promote calcification, resulting in aggregation of hydroxyapatite crystals. Calcification in the valves does not form in circular lamellae, but has a disorganized pattern, indicating dystrophy similar to that seen in atherosclerotic plaque maturation. As collagen is degraded it is replaced with calcium hydroxyapatite, making the valve stiff and inflexible, with possibility of rupture [80].

### 4.3. ECM Components as Signaling Mediators in Heart Valves

The valve ECM is rich in biochemical molecules and can regulate various cellular and biological processes [96], and thus, its dysregulation is often associated with altered cell signaling. The influence of ECM on physiological cellular functions can be broadly classified into three types: (i) matricellular signaling, (ii) matricrine signaling, and (iii) mechanical signaling (Figure 2) [97].

In matricellular signaling, the ECM components regulate active intracellular processes through their interactions with adhesion receptors, such as integrins, other ECM proteins and cell surface receptors [98]. Smaller ECM proteins, such as fibronectins and laminins aide in this cross-talk between different ECM components that subsequently result in a physical frame work necessary for the matricellular signaling [98,99,100]. Adhesion receptors, such as those belonging to the integrin family play a prominent role in matricellular signaling by establishing the cross-talk between the ECM components and cells [50]. Integrins, the heterodimeric transmembrane receptors, with a large extracellular domain, are activated by the matrix ligands. Subsequently, activated integrins initiate signaling cascade of events mediated by focal adhesion kinases (FAK) and integrin-linked kinases (ILK) [50,98]. In human heart valves, previous studies have identified expression of Integrin α-1, α-2, α-3, α-4, α-5, and α-11 at different levels in the VICs from all the leaflets [101]. Additionally, the Integrin α2β1 heterodimer has been shown to mediate binding of VICs to ColI leading to VIC contraction and leaflet force generation [42]. Decorin, has been reported to activate the TGF-β signaling pathway, through matricellular signaling, by promoting TGF-β production [102].

In matricrine signaling, the ECM components particularly PGs, sequester growth factors and cytokines, leading to ECM serving as a reservoir, often at a gradient concentration for several cellular signals [50,62,97,98,103]. Additionally, ECM components play a role in maturation of inactive ligands such as latent TGF-β, that is stored in ECM upon secretion until proteolytically activated by the MMPs (Figure 2B) [50,104]. In vascular smooth muscle cells, CS or DS, through a matricrine-signaling mechanism, increase bioavailability of TGF-β resulting in activation of osteogenic differentiation pathways [102]. However, it remains to be studied whether they play a similar function in heart valves as well. Additionally, PGs such as biglycan and decorin have been implicated in accelerating valve calcification by localizing TGF-β, TNF-α, or other growth factors associated with calcific aortic valve disease (CAVD), to the calcific nodules in the aortic valve [37]. GAGs, such as hyaluronan have also been implicated in facilitating matricrine signaling by mediating interactions between ligands and their receptors [97]. HS-GAG has been reported to form a multimeric complex with fibroblast growth factor (FGF) and its receptor that promotes FGF signaling [97,105].

In mechanical signaling, the valve ECM regulates the cellular functions in response to changes in its intrinsic features, such as matrix elasticity or stiffness and other changes in the microenvironment. Several in vitro studies have identified the effect of ECM stiffness on the VIC behavior and functions [106]. In valves, ECM stiffness has been reported by multiple studies to regulate VIC phenotypes and differentiation [107,108,109,110,111]. In human aortic VICs, altered matrix stiffness has been reported to drive the cells towards pathogenic differentiation [108]. Studies using the porcine aortic valve model further demonstrate that VICs, grown on a rigid collagen matrix, leads to enhanced myofibroblast differentiation [109] and that matrix stiffness can influence the response of porcine VICs to pro-calcific factors [110], suggesting the importance of changes in ECM in valve pathologies, such as CAVD and myxomatous valve disease. Additionally, changes in ECM substrate composition also resulted in altered response of VICs to pathogenic stimuli [107]. Interestingly, the effect of ECM stiffness on the valve cells seems to be dependent on age and valve leaflet regions [44]. During the cardiac cycle, valves are exposed to several forces such as hemodynamic shear stress, stretch and pressure and ECM transmits these external signals to the cells and regulate valve cell phenotype and processes such as gene expression and protein activation [112]. Altered biomechanics in valve cells, such as cyclic strain results in modulation of inflammatory cytokine expression in both interstitial and endothelial cells in vitro [113,114]. Ex-vivo studies using porcine aortic valves have demonstrated that increased cyclic stretch can regulate the expression of bone morphogenic proteins resulting in valve calcification [115]. In addition, ECM has also been implicated in affecting cell proliferation, polarity and contractility [50], denoting the key role of ECM in regulating valve cell signaling and processes.

## 5. ECM and Heart Valve Disease

Clinically important heart valve pathology most frequently involves a disruption in normal structure-function correlations by genetic, mechanical, and inflammatory/immunological factors, through abnormal and complex interaction of VECs, VICs, ECM and their environment. Heart valve disease can be congenital, referring to phenotypes present at birth, and includes structural malformations, such as bicuspid aortic valve. Advancements in human genome sequencing have led to identification of genetic causes involved in the formation of congenital lesions in utero. Alternatively, heart valve disease can be acquired and manifested later in life, and includes calcification and myxomatous degeneration [116]. The underlying cause of acquired valve disease is complex and thought to be the result of age-related degeneration following life-long exposure to known risk factors, including male gender, hypertension, and smoking causing “wear and tear” stress on the leaflets over the course of a lifetime [65]. Additionally, inflammatory mechanisms are increasingly thought to play a role in valve disease. Alternatively, though some cases are still considered idiopathic, there is emerging data to suggest that acquired valve disease has origins during embryonic development, but phenotype development is delayed, and likely requires additional risk factors to be present.

### 5.1. Histological Characteristics of Diseased, or Dyfunctional Heart Valves

As discussed in detail, stratification of the valve ECM components within the healthy valve is essential in maintaining proper function, as each layer provides a unique and essential biomechanical purpose to tolerate a lifetime of hemodynamic forces [2,65]. In contrast to this intricate architecture, histological examinations of end-stage disease valve tissue at the time of surgical replacement, commonly report aberations in the organization and composition of the ECM layers [8,117,118]. This includes a breakdown of otherwise healthy ECM, likely due to excess activation of proteinases (e.g., MMPs) or reduced activity of their inhibitors, and an over-, or under-abundance in the secretion of key ECM components. The pathological change in the ECM is disease-specific and dictates the clinical and functional phenotype [8]. For example, excess PG deposition and fragmentation of collagen fibers leads to a myxomatous phenotype, which in the mitral position, forces the leaflet to prolapse, or abnormally bulge back into the left atrium. In contrast, abnormal mineralization of the valve ECM promotes the formation of calcific nodules on the leaflet surface, leading to a stiffened and less compliant tissue, resulting in stenosis [8]. Therefore, the degree of pathological ECM remodeling dictates the extent of dysfunction, which is further compromised by gross thickening of the tissue as the ECM expands.

### 5.2. Cellular Changes in Diseased, or Dysfunctional Heart Valves

While the underlying mechanisms that govern ECM alterations and subsequent valve dysfunction in affected individuals are not known, although, VICs are thought to play a role. As mentioned previously, in healthy valves, VICs are quiescent as noted by their lack of SMA expression. However, SMA-positive aVICs are present in most forms of acquired valve disease, while less is known in congenital cases. The contribution of VICs to ECM pathogenesis in adult valves is not clear, but studies have shown co-localization with MMPs and other matrix remodeling enzymes, as well as regions of ECM remodeling [8,9,62,119]. SMA-positive aVICs are also observed through stages of physiological ECM remodeling during valvulogenesis, and therefore it has been postulated that aVICs play a similar role in mediating matrix changes in disease. However, this process is less controlled. While endothelial-to-mesenchymal transitions have been shown to give rise to SMA-positive cells in the embryo, fate map studies in valve disease models suggest that this process does not occur in adult valve disease [120], but it may mediate disease in canine models [121]. As there is accumulating data to suggest that the transition of qVICs to aVICs may be one of the early triggers that initiates progressive degeneration, the field has turned to understanding the mechanistic triggers. Despite these cells being identified over 25 years ago, the phenotypic switch is poorly understood, but insights from in vitro assays have shown that TGF-β1 and mechanical stiffness are positive regulators, while the actin binding protein cofilin negatively regulates VIC activation [65,66,108,109,110]. Studies by the Bischoff group also showed that the presence of VECs, prevents activation of cultured VICs through a currently unknown paracrine signal [122]. Therefore, it is suggested that this inhibitive effect of VECs on VIC activation may be compromised in valve disease, associated with underlying VEC dysfunction. These collective studies suggest that maintaining VIC quiescence in healthy valves is complex and likely multi-factorial, and additional work is needed to fully understand this process in a disease-dependent manner.

### 5.3. Connective Tissue Disorders and Heart Valve Disease

Mutations in ECM components, such as fibrillin, elastin, or collagen, cause connective tissue disorders, which are multisystemic diseases and affect tissues, including joints, bones, skin, eyes, lungs, and the cardiovascular system, including heart valves [123,124,125]. To better understand the pathogenesis of these diseases, mouse models have been developed to recapitulate phenotypes seen in some of the more common connective tissue disorders in humans, including Marfan syndrome (MS), Ehlers-Danlos Syndrome (EDS), and Osteogenesis Imperfecta (OI). As discussed below and highlighted in Table 1, mouse models of these human mutations have shed mechanistic insights into syndromic valve disease pathobiology.

Mutations in *fibrillin-1* cause MS, [123] an autosomal-dominant disorder affecting roughly one in 5000 individuals worldwide [126]. MS is characterized by skeletal abnormalities, joint laxity, and ocular lens dislocation [127,128]. However, the cardiovascular manifestations, such as aortic dissection and mitral and aortic valvular dysfunction, are the most common cause of reduced life expectancy [21], and can be present at birth or acquired later [129,130]. It has been reported that mitral valve prolapse affects as many as 77% of MS patients by 60 years of age [131]. These cardiovascular phenotypes are recapitulated in the *Fbn1^C1039G/+^* mouse model [127], with mice developing myxomatous mitral valve features, including leaflet thickening, increased PG deposition, and collagen fragmentation, by 2 months of age [130] (see Table 1).

EDS is categorized into 13 different subtypes based on a spectrum of symptoms including joint hypermobility, skin hyperextensibilty, muscle hypotonia, arterial aneurysm, skeletal abnormalities, gastrointestinal complications, and occular problems [132,133,134]. EDS has been linked to mutations in at least 19 different genes [132], including *ColVa1*, which has been identified in over 40% of EDS cases and can be modeled in mice [20]. While, *ColVa1*-/- mice die at embryonic day 10.5 [20], *ColVa1+/-* mice are a viable model of several phenotypes seen in EDS, including decreased tensile strength of the aorta, hyperextensible skin [135], and when crossed with *ColXIa1* mutant mice, show increased atrioventricular valve thickness compared to wildtype mice [20] (see Table 1).

OI, also known as brittle bone disease, is most commonly caused by mutations in the *ColIa1* or *ColIa2* chains of type I collagen [136]. It affects approximately 1 in every 10,000 to 20,000 births, but varies greatly in severity, ranging from mild to perinatally lethal [136]. OI can be classified into at least 15 different types [137], most of which are caused by autosomal dominant mutations, though some less common types are autosomal recessive or X-linked recessive [138]. Mice carrying a single G deletion in the proα2 chain of collagen I were first described as a model of OI in 1993 [139]. They named the mutation osteogenesis imperfecta murine (OIM), and described progressively severe skeletal abnormalities, present in the homozygous mutants, which are identifiable at birth by their “drooping wrist” phenotype and “appearance of hemorrhages into joint cavities, sides of the body, or around the scapulas” [139]. These mice have since been described as having abnormalities of the heart valves as well, showing progressive distal thickening and increased PG deposition in the aortic valve leaflets by 5 months of age [117]. Aortic and mitral valve regurgitation has been seen in humans with OI [140,141,142], though valvular dysfunction at least by 9 months of age, has not yet been reported in these mice.

## 6. ECM and Therapeutics

Approximately 10,000 deaths per year are directly attributed to heart valve disease in the United States. There are no medical therapies for preventing or slowing the progression of valvular pathology. Our lack of medical options arises from our limited understanding of the cellular and molecular mechanisms underlying heart valve disease, and how abnormalities at these scales propagate to valve-level function. Improving our understanding of the factors, which govern ECM production and remodeling, holds the key to understanding heart valve function and pathological dysfunction. Furthermore, understanding the mechanobiological principles that govern heart valve development, growth and remodeling will be critical to developing effective medical therapies. As we begin to elucidate the cellular and molecular mechanisms underlying heart valve disease, we should be able to rationally design therapeutics to prevent, abate or even reverse progressive heart disease. Based on the structure-function correlate, the function of the heart valve is determined by its mechanical properties, which in turn, are dictated by the biomechanical properties of the ECM.

Currently, the only long-term effective treatment for heart valve disease is heart valve repair or replacement. This area of research has been extensively reviewed elsewhere, [149], but in brief, approximately 60,000 heart valve replacements are performed per year in the United States. Since the 1960s, heart valve substitutes have undergone a progressive evolution. Many modifications have been made and new designs have been introduced to address specific deficiencies in earlier devices. Currently available valve replacement devices include mechanical heart valves and biological heart valves. Each type of substitute valve is associated with its own unique set of complications. Within 10-years of initial heart valve replacement, over 50% of patients experience prosthesis-associated problems, which require reoperation. The overall rate of complications is similar for mechanical prostheses and bio-prostheses. Four categories of valve related complications predominate: (i) thromboembolism, thrombosis, secondary anticoagulation related hemorrhage; (ii) prosthetic endocarditis; (iii) structural dysfunction including failure or degeneration of the prosthetic biomaterials; and (iv) non-structural dysfunction including complications arising from technical problems during surgical implantation such as peri- valvular leak and biological integration (tissue overgrowth) into the host.

The development of a tissue engineered heart valve (TEHV) created from an individual’s own cells with the ability to grow, repair, and remodel offers a potential solution to this problem. Significant progress has been made including the performance of the first clinical trials evaluating the safety of this technology [150,151,152]. Unfortunately, the preliminary results of these trials have demonstrated some significant valve-related complications that have prevented their widespread use, most notably leaflet retraction which leads to regurgitation. Currently available TEHV can broadly be separated into two categories, based on the scaffold used to create the TEHV [153]. Synthetic scaffolds are typically fabricated from man-made synthetic polymers including a variety of biomaterials such as polyglycolic acid, polycaprolactone, and 4-hydroxybuterate. Alternatively, ECM-based scaffolds can be fabricated from either decellularized xenograft or homograft valves using either detergent-based or enzymatic methods. The scaffolds can be either unseeded or seeded with autologous cells including differentiated cells such as EC, myofibroblasts, or VICs, stem cells, or alternative cell sources including bone marrow-derived mononuclear cells. The seeded constructs can be developed ex vivo, using a bioreactor or the seeded construct can be implanted shortly after seeding, allowing the bulk of neotissue formation to occur in vivo. The optimum design of a TEHV has yet to be achieved but perhaps the most clinically advanced TEHV have utilized in situ tissue engineering techniques in which unseeded scaffold is used to induce autologous neotissue formation. The scaffold initially functions as the replacement heart valve but over time serves as a template as the neotissue forms and gradually replaces the scaffold.

The key to optimizing the design of the TEHV is to improve our understanding of the factors which control neotissue formation in TEHV. Neotissue formation appears to result from the interplay of genetic factors, inflammation, and mechano-mediated remodeling. Understanding the cellular and molecular mechanisms that drive ECM production would enable us to rationally design an improved TEHV. The development of model systems enable us to determine how these factors affect neotissue formation in isolation and represents an important first step. This should be followed by the development of computational models, which when properly informed by the experimental data, can be used to determine the complex interplay of the factors. Ultimately the design of the scaffold can be used to direct neotissue formation and optimize the performance of the TEHV.

## 7. Conclusions

In closing, this review highlights the key ECM components that form the three-dimensional network within the valve structures and offer structural and biochemical support to the valve cell populations, in addition to providing all the necessary biomechanical properties needed to repetitively open and close. At present, the availability of effective, long-lasting therapies in the treatment of heart valve disease are limited and it is critical that alternatives are found in order to improve patient outcome. Here, we reviewed the key components that are needed to maintain structure-functional relationships of the heart valve and identified how the dynamic orchestration of this ECM niche regulates valve phenotypes and influences response. As the field continues to grow, we are learning more about the ‘triggers’ that initiate ECM disturbances in disease through the development of molecular approaches, imaging, computational remodeling and tissue engineering approaches. Through this multi-disciplinary approach, we are becoming poised to further advance our understanding of the fine balance required for maintaining heart valve homeostasis and the development of mechanistic-based therapies beyond surgical intervention.

## Figures and Tables

**Table 1:** Mouse models of human connective tissue disorders with valvular defects.

Human Disease	Associated ECM Gene	Mouse Model	Human Valvular Defects Recapitulated in the Mouse Model	References
Williams-Beuren Syndrome	*Elastin*	Deletion (from Gtf2i to Fkbp6) (includes ELN) (most common deletion found in humans with WBS)	Supravalvular aortic stenosis (SVAS)	[125]
Loeys-Dietz Syndrome	*TGF-* *βR1* *TGF-* *βr2R2* *TGF-* *β1* *SMAD3*	Loss-of-function mutations: *-Tgfβr1^M318R/+^*^*−*^*Tgfβr2^G357W/+^* -Transgenic *Tgfβr2*	Aortic aneurysm Mitral valve prolapse	[143] [144] [145] [146]
Cutis Laxa	*FBNL4* *FBNL5*	*Fibulin-4^R/R^* (reduced expression allele)	Thickened aortic valve leaflets with stenosis and insufficiency	[145] [146]
Marfan Syndrome	*FBN1*	*Fbn1^C1039G/+^*	Mitral valve prolapse	[127]
Ehlers-Danlos Syndrome	*COLVA1*	*ColVa1^+/−^*	No gross of functional valve defects	[20]
Stickler Syndrome	*COLXIA1*	*ColXIa1^−/−^*	Thickened valve leaflets	[20]
Osteogenesis Imperfecta	*COL1A2*	*Col1a2^−/− (oim/oim)^*	Myxomatous aortic valve leaflets	[117]
Fibrotic aortic valve disease-autosomal-dominant connective tissue disease	*EMILIN-1*	*Emilin1^−/−^*	Marked progression of valve pathology that shows severe fibrosis, neovascularization and inflammation	[147] [148]
